# Overcoming endocrine resistance in metastatic hormone receptor-positive breast cancer

**DOI:** 10.1186/s13045-018-0620-6

**Published:** 2018-06-11

**Authors:** Anishka D’Souza, Darcy Spicer, Janice Lu

**Affiliations:** USC Norris Comprehensive Cancer Center, 1441 Eastlake Avenue, Los Angeles, CA 90033 USA

## Abstract

Endocrine therapy has historically formed the basis of treatment of metastatic hormone receptor-positive breast cancer. The development of endocrine resistance has led to the development of newer endocrine drug combinations. Use of the CDK4/6 inhibitors has significantly improved progression-free survival in this group of patients. There are multiple studies of the use of P13K inhibitors and mTOR inhibitors for use as subsequent lines of therapy, particularly for endocrine resistance. The optimal sequencing of therapy should be based on medical comorbidities, prior adjuvant therapies, quality of life, side-effect profile, and disease-free interval.

## Background

Breast cancer is the most frequently diagnosed cancer in women. Around 5–10% of cases are metastatic at diagnosis, and close to 30% of patients with early stage disease will go on to relapse with metastatic disease [1]. Hormone receptor-positive breast cancer makes up 70% of breast cancers cases. Endocrine therapy remains the mainstay of early treatment. A significant number of these patients will develop either primary or secondary endocrine resistance, prompting the need for newer treatment options [2].

### Endocrine therapies

Tamoxifen has been used in the management of metastatic hormone receptor-positive breast cancer for decades. The third-generation aromatase inhibitors (AIs) are used in both the first- and second-line settings in the management of hormone receptor-positive metastatic breast cancer.

Fulvestrant is an selective estrogen receptor downregulator (SERD) used in the management of metastatic hormone receptor-positive breast cancer in both the first- and subsequent-line settings. The 500 mg fulvestrant dose was approved based on the results of the CONFIRM trial, which showed improvement in both progression-free and overall survival with the 500-mg dose compared with the 250-mg dose [3]. The FIRST trial compared the use of fulvestrant 500 mg monthly with anastrazole 1 mg daily in postmenopausal women with advanced or metastatic hormone receptor-positive breast cancer. This study demonstrated a significant improvement in time to progression and an improved overall survival in the fulvestrant compared with the anastrazole group [4, 5]. The FALCON trial further assessed the progression-free survival advantage observed in the FIRST study. This was a phase III study comparing the use of fulvestrant 500 mg monthly with anastrazole 1 mg daily in endocrine therapy-naïve, postmenopausal patients with metastatic hormone receptor-positive breast cancer [6]. A total of 462 patients were randomized to treatment. Median progression-free survival was 16.6 months with fulvestrant and 13.0 months with anastrazole (*P* = 0.0486). The objective response rate in patients with measurable disease was 46 and 45%, respectively, in patients treated with fulvestrant and anastrazole. The median duration of response was longer in the group treated with fulvestrant (20.0 months) compared with that in the anastrazole group (13.2 months). Data is as yet immature for evaluation of overall survival. The rates of adverse events were similar in the fulvestrant (73%) and anastrazole (75%) groups, with the rates of serious adverse events in both groups being 13%. Overall, the results of the FALCON study support the superior efficacy of fulvestrant over anastrazole in the treatment of postmenopausal women with hormone receptor-positive metastatic breast cancer who did not receive previous endocrine therapy. In addition, patients with disease confined to the bone tended to do better with fulvestrant therapy. Fulvestrant is FDA-approved for use in both endocrine-resistant and naïve settings.

### Resistance to endocrine therapy

Endocrine resistance is a major clinical problem encountered in the treatment of breast cancer. Endocrine resistance can be divided into two groups. Primary endocrine resistance is defined as relapse during the first 2 years of adjuvant endocrine therapy or progressive disease within the first 6 months of first-line endocrine therapy for metastatic breast cancer. Secondary resistance is defined as relapse while on adjuvant endocrine therapy but after the first 2 years of treatment, relapse within 12 months of completing adjuvant endocrine therapy, or progressive disease six or more months after starting endocrine therapy for metastatic breast cancer [7].

There are a number of different mechanisms implicated in the development of endocrine resistance. Loss of estrogen receptor (ER) expression is one possible cause, with between 10 and 20% of initially ER-positive patients converting to negative on relapse [8]. Another mechanism being explored is the development of endocrine receptor mutations [9]. Variations in tamoxifen metabolism have been studied as a possible cause of differing responses to tamoxifen therapy. The CYP2D6 gene product is responsible for the metabolism of tamoxifen to endoxifen, one of its active metabolites. The drug metabolism varies based on the CYP2D6 allelic profile, with certain genotypes associated with a higher rate of relapse [8]. Growth factors, such as EGFR, insulin/IGFs, and FGFR, and their respective signaling pathways have also been studied as potential mechanisms of endocrine resistance [9]. Studies suggest that HER2/neu overexpression is associated with poorer response to tamoxifen therapy. HER2 may lead to tamoxifen resistance by activating estrogen receptor co-activator proteins [8]. P13K/AKT/mTOR signaling pathway activation plays a major role in the development of endocrine resistance and is a target of many therapies designed to overcome resistance [10]. There are also studies evaluating the role of the ESR1 mutation in acquired endocrine resistant breast cancer. There is an ongoing phase II study evaluating the efficacy of fulvestrant in patients with ESR1-mutated breast cancer (NCT03202862).

### CDK4/6 inhibitors

#### Mechanism of action

Cyclin-dependent kinases (CDKs) 4 and 6 promote entry into the cell cycle through the phosphorylation of the retinoblastoma protein (Rb) [11]. This leads to the transition from gap 1 (G1) to the DNA synthesis (S) phase of the cell cycle, ultimately leading to cell division [12]. Activation of the pathway leading to Rb phosphorylation has been associated with the development of endocrine resistance [10]. CDK 4/6 inhibitors block the phosphorylation of Rb, leading to cell cycle arrest, and can reverse endocrine resistance when used. Three CDK 4/6 inhibitors currently being used in the treatment of advanced breast cancer are palbociclib, ribociclib, and abemaciclib (Table 1) [13, 14].

**Table 1:** Summary of trials using CDK4/6 inhibitors in patients with metastatic hormone receptor-positive breast cancer

Agent	ET/Setting	Median PFS	HR	Trial
Palbociclib	Letrozole, 1st-line	24.8 vs 14.5 months	0.58	PALOMA-2
Palbociclib	Fulvestrant, 2nd-line	9.2 vs 3.8 months	0.42	PALOMA-3
Ribociclib	Letrozole, 1st-line	25.3 vs 16.0 months	0.56	MONALEESA-2
Abemaciclib	Fulvestrant, 2nd-line	16.4 vs 9.3 months	0.55	MONARCH-2

*ET* endocrine therapy, *PFS* progression-free survival

#### Palbociclib

Palbociclib is an oral, selective inhibitor of CDK 4/6 approved for use in the first- and second-line settings for advanced or metastatic hormone-receptor positive breast cancer.

PALOMA-2 was a phase III study of palbociclib and letrozole as first-line therapy for postmenopausal women with estrogen-receptor (ER)-positive, HER2-negative advanced breast cancer [15]. A total of 666 women were randomly assigned, in a 2:1 ratio, to receive either palbociclib 125 mg administered in 4-week cycles (3 weeks on, 1 week off) or placebo, in combination with continuous daily letrozole 2.5 mg. The median age of patients was 62 years in the palbociclib-letrozole group and 61 years in the placebo-letrozole group. Of all patients, 37.2% had newly diagnosed metastatic breast cancer, 40.7% had a disease-free interval of more than 12 months, and 22.1% had a disease-free interval of less than 12 months. The median progression-free survival was 24.8 months in the palbociclib group and 14.5 months in the control group (HR 0.58; 95% CI, 0.46–0.72; two-sided *P* < 0.001). Data from longer-term follow-up of the PALOMA-2 trial were presented at the 2017 San Antonio Breast Cancer Symposium and revealed a median PFS of 27.6 months in the palbociclib/letrozole arm at 38 months follow-up and 14.5 months in the placebo arm at 37 months follow-up [16]. The objective response rates among all patients randomly assigned to receive palbociclib-letrozole were 42.1% (95% CI, 37.5–46.9) compared with 34.7% (95% CI, 28.4–41.3) in patients randomized to receive placebo. At the time of publication, the overall survival data was still immature. The most common adverse events in the study group were neutropenia, leucopenia, fatigue, nausea, arthralgia, and alopecia. Grade 3 or 4 hematologic events included neutropenia (66.4% of patients in palbociclib-letrozole group vs. 1.4% of patients in the placebo-letrozole group), leucopenia (24.8 vs. 0%), anemia (5.4 vs. 1.8%), and thrombocytopenia (1.6 vs. 0%). Nonhematologic adverse events included fatigue, nausea, and arthralgia.

PALOMA-3 was a phase III study of the use of palbociclib in previously treated patients with advanced hormone-receptor positive, HER2-negative breast cancer [11]. Patients were eligible if their cancer had relapsed or progressed with prior endocrine therapy. Both pre- and postmenopausal women were allowed to participate. A total of 521 patients were randomly assigned in a 2:1 ratio to receive palbociclib (125 mg daily 3 weeks on and 1 week off cycles) or matching placebo in addition to fulvestrant (500 mg every 14 days for 3 doses followed by every 28 days thereafter). The median age of all patients was 57 years, with the majority of patients (79.3%) being postmenopausal. Of all patients enrolled, 23.4% had metastatic disease at initial diagnosis. The median progression-free survival was 9.2 months in the palbociclib-fulvestrant group and 3.8 months in the placebo-fulvestrant group (HR 0.42; 95% CI, 0.32–0.56; *P* < 0.001). The relative differences in PFS were similar between the pre- and postmenopausal groups. The objective response rate was higher in the study versus the placebo group; however, this difference was not statistically significant (10.4 vs. 6.3%, *P* = 0.16). Grade 3 or 4 hematologic toxicities were all more frequent in the palbociclib-fulvestrant group when compared with the placebo-fulvestrant group (neutropenia 62.0 vs. 0.6%, leucopenia 25.5 vs. 0.6%, anemia 2.6 vs. 1.7%, and thrombocytopenia 2.3 vs. 0%). The most frequent nonhematologic toxicities were fatigue, nausea, and headache.

The PEARL study is a phase III trial comparing the use of palbociclib combined with endocrine therapy (exemestane or fulvestrant) with chemotherapy (capecitabine) in patients with HR+/HER2- metastatic breast cancer who are resistant to a nonsteroidal aromatase inhibitor [17].

The POLARIS trial is a prospective, non-interventional study of 1500 patients who were treated with palbociclib in the USA and Canada. The study will evaluate the prescribing and treatment patterns of the patients; assess the clinical response to palbociclib; perform biomarkers studies to help elucidate potential mechanisms of response and resistance to palbociclib; assess patient quality of life, study survival, and toxicity of the drug; and evaluate sequencing of treatments in these patients [18].

#### Ribociclib

Ribociclib (LEE011; Novartis) is a selective inhibitor of CDK 4/6 currently being studied for use in the treatment of pre- and postmenopausal women with advanced hormone-receptor positive breast cancer.

Based on the results of the phase III MONALEESA-2 trial, the FDA approved its use in combination with letrozole as first-line endocrine therapy in postmenopausal patients with hormone receptor-positive breast cancer [19]. Patients were postmenopausal with locally recurrent or metastatic hormone receptor-positive, HER2-negative breast cancer who had not previously received systemic therapy for their advanced disease. A total of 668 patients were randomized to receive ribociclib (600 mg per day on a 3-week on and 1-week off schedule) plus letrozole (2.5 mg per day continuously) or placebo plus letrozole. The median age of patients was 62 years, with 34.0% having advanced or metastatic disease at initial diagnosis. After a median follow-up of 26.4 months, the median progression-free survival was 25.3 months in the ribociclib group and was 16.0 months in the placebo group (HR 0.56; 95% CI, 0.43–0.72; *P* = 3.29 × 10^−6^). At 12 months, the progression-free survival was 72.8% in the ribociclib group and 60.9% in the placebo group. At 18 months, the progression-free survival was 63.0 and 42.2%, respectively. The overall response rates were 40.7% in the ribociclib group and 27.5% in the placebo group in the intention-to-treat population. At the time of the interim analysis, overall survival results were not mature. Grade 3 or 4 adverse events included neutropenia (59.3% in the ribociclib group vs. 0.9% in the placebo group), leucopenia (21.0 vs. 0.6%), hypertension (9.9 vs. 10.9%), and elevated alanine aminotransferase level (9.3 vs. 1.2%). QTc interval prolongation of at least 60 msec from baseline occurred in 9 patients in the ribociclib group (2.7%) and no patients in the placebo group.

The MONALEESA-3 trial (NCT02422615) is currently ongoing. This study is evaluating the use of ribociclib in combination with fulvestrant in patients with advanced breast cancer who have received one prior line of endocrine therapy.

The phase III MONALEESA-7 trial (NCT02278120) is specifically designed for pre- or perimenopausal women with advanced hormone receptor-positive breast cancer. Patients were randomized to receive tamoxifen or a non-steroidal aromatase inhibitor (letrozole or anastrozole) with goserelin in combination with ribociclib or with placebo. Progression-free survival was significantly improved in the ribociclib arm (23.8 months) versus the placebo arm (13.0 months) [20].

#### Abemaciclib

Abemaciclib (LY2835219) is the third CDK 4/6 inhibitor currently being studied for use in hormone receptor-positive advanced breast cancer. It is unique from palbociclib and ribociclib in that it can be dosed continuously and has a higher response rate when used as monotherapy [12, 13]. It also demonstrates greater selectivity for CDK4 than for CDK6. In the phase I study investigating the use of the drug in patients with various solid tumors, the dose-limiting toxicity was diarrhea, which is different from the DLT of bone marrow suppression seen with the other CDK4/6 inhibitors [21]. Abemaciclib was recently FDA-approved for the use in combination with fulvestrant in the management of hormone receptor-positive, HER2-negative metastatic breast cancer after progression on endocrine therapy. It is also approved as monotherapy in patients with hormone receptor-positive/HER2- metastatic breast cancer who had progressed during or after treatment with endocrine therapy and chemotherapy.

The use of abemaciclib monotherapy is based on the results of the MONARCH-1 trial [22]. This was a phase II, single-arm study of the use of abemaciclib monotherapy in women with hormone receptor-positive, HER2-negative metastatic breast cancer who have progressed on endocrine therapy or chemotherapy. A total of 132 patients were treated with abemaciclib monotherapy on the study, with a median of 3 prior lines of therapy for advanced disease. At the 8-month interim analysis, the overall response rate was 17.4% and the median progression free survival was 5.7 months.

Support for the use of the abemaciclib and fulvestrant combination came from the results of the phase III MONARCH-2 study [23]. This was a randomized trial comparing the use of fulvestrant with or without abemaciclib in patients with hormone receptor-positive, HER2-negative advanced breast cancer who had progressed on endocrine therapy. A total of 669 patients were randomized. The median progression-free-survival was 16.4 months in the combination group, compared with 9.3 months in the fulvestrant-only group (HR, 0.553; 95% CI, 0.449–0.681; *P* < .001). The overall response rate was 35.2% in the abemaciclib group, with 14 patients (3.1%) attaining a complete response. This compared with an ORR of 16.1% in the control arm and one CR (0.4%). Overall survival data was not yet mature at the time of data cutoff. The most commonly occurring adverse events were neutropenia, diarrhea, fatigue, nausea, and abdominal pain, the majority of which were grade 1 or 2. Serious adverse events occurred in 22.4% of patients in the combination arm and 10.8% of patients in the control group.

The MONARCH-3 study evaluates the use of abemaciclib with letrozole as first-line treatment in postmenopausal women with metastatic hormone receptor-positive breast cancer [24]. Results of the trial interim analysis were presented at ESMO this year and demonstrated a significant improvement in progression-free survival with a hazard ratio of 0.543 (*p* = 0.000021). The objective response rate in patients with measurable disease was 59% in the abemaciclib arm compared with 44% in the control arm.

#### CDK4/6 resistance

Proposed mechanisms of resistance to CDK4/6 inhibitors include the MAPK and PI3K pathways, mutations or deletions of RB1, and amplification or overexpression of cyclin E1.

### mTOR inhibitors

The PI3K/AKT/mTOR pathway is dysregulated in many types of cancers. It is regulated at different points by multiple tumor suppressor and oncogenes, which provide sites of potential mutations in the pathway [25]. P13K alterations are common in many cancers, with activating mutations of the P1K3CA gene commonly found in breast cancer. Other common mutations in the pathway found in breast cancer include mutated AKT and loss of PTEN [26]. Dysregulation in the mTOR pathway have been implicated in the development of endocrine resistance in breast cancer, making this an attractive target for therapy [2]. Everolimus is a rapalogue or rapamycin analogue that acts as a potent inhibitor of mTOR. It has been the focus of a variety of trials for the use in hormone receptor-positive breast cancer.

The TAMRAD study was a phase II trial evaluating the use of everolimus in combination with tamoxifen in postmenopausal women with metastatic hormone receptor-positive, HER2-negative breast cancer who had progressed on prior aromatase inhibitor therapy [27]. The clinical benefit rate at 6 months was 61% in the everolimus arm and 42% in the tamoxifen only arm (*P* = 0.045). The time to progression was also significantly improved in the everolimus group (8.6 vs. 4.5 months; *P* = 0.002). There was a 46% reduction in the risk of progression and 55% reduction in the risk of death associated with the everolimus-tamoxifen combination. On subgroup analysis, it appeared that the benefit of the combination therapy was mainly for patients who had secondary endocrine resistance.

The BOLERO-2 study evaluated the use of an everolimus-exemestane combination in the treatment of hormone receptor-positive, HER2-negative metastatic breast cancer [28]. In 2012, the FDA approved the use of the combination in this group of patients who had progressed on prior aromatase inhibitors. In the phase III trial, postmenopausal women with ER-positive, HER2-nonamplified advanced breast cancer who had progressed on prior aromatase inhibitors were randomized in a 2:1 ratio to receive oral everolimus (10 mg daily) or placebo, in combination with exemestane (25 mg daily). A total of 724 women were randomized, with a median age of 62 years. Fifty-six percent of the patients had visceral involvement of their cancer and 76% had bone metastases. The median progression-free-survival was 6.9 months in the everolimus arm and 2.8 months in the placebo arm (HR 0.43; 95% CI, 0.35–0.54; *P* < 0.001). Median overall survival was not significant between the two groups (31.0 months in the everolimus-exemestane group and 26.6 months in the placebo-exemestane group; *P* = 0.1426 [29]. The most common grade 3 or 4 adverse events were stomatitis, dyspnea, hyperglycemia, fatigue, and pneumonitis, all of which were more common in the everolimus arm.

The BOLERO-4 study (NCT01698918) is a currently ongoing trial evaluating the use of everolimus in combination with letrozole as first-line therapy for postmenopausal women with hormone receptor-positive, HER2-negative metastatic breast cancer.

The BALLET trial was a single-arm European phase IIIb study evaluating the safety of everolimus plus exemestane in post-menopausal women with hormone receptor-positive, HER2-negative advanced or metastatic breast cancer who had progressed on prior non-steroidal aromatase inhibitors [30]. A total of 2133 patients were enrolled in the study and were treated with daily doses of everolimus (10 mg per day) and exemestane (25 mg per day). The median age of patients enrolled was 63 years. The majority of patients (65%) received everolimus/exemestane as a third or higher line of therapy. Overall, 42.7% of patients experienced grade 3/4 adverse events, with the majority of these being attributed to the everolimus. At the time of study analysis, 121 (5.7%) on-treatment deaths were documented, with 66 (3.1%) of these attributed to disease progression and 46 (2.2%) attributed to adverse events.

The MANTA trial is a four-arm, phase II study of fulvestrant + continuous AZD2014 (vistusertib), fulvestrant + intermittent vistusertib, fulvestrant + everolimus, and fulvestrant alone in women with estrogen receptor-positive, HER2-negative advanced or metastatic breast cancer [31]. AZD2014 is a novel inhibitor of mTORC1 and mTORC2. The everolimus/fulvestrant arm demonstrated improved PFS compared with vistusertib/fulvestrant (12.3 vs 7.6 months) and with fulvestrant alone (12.3 vs 5.4 months). The objective response rate was also higher in the everolimus/fulvestrant group (41%) when compared with the fulvestrant alone (25.0%), fulvestrant/vistusertib continuous (30.4%), and fulvestrant/vistusertib intermittent (28.6%) groups.

### HR+ and HER2+

For HER2/HR-positive patients, treatment with HER2-targeted therapy in combination with chemotherapy is the most common first-line therapy. There are no trials directly comparing the use of endocrine plus HER2-targeted therapy with the use of chemotherapy combined with HER2-targeted agents. Treatment with a HER2-targeted agent in combination with endocrine therapy can be considered in certain patients, in whom treatment with chemotherapy is not immediately indicated.

Studies of mouse models indicate that the cyclin D1-CDK4 pathway may play a role in the development of resistance to HER2-directed therapies in patients with HER2-positive breast cancer. The use of CDK4/6 inhibitors in this population may resensitize these patients to anti-HER2 therapy. The monarcHER study is a randomized, multicenter, phase 2 trial comparing the use of abemaciclib and trastuzumab with or without fulvestrant to physician’s choice standard-of-care chemotherapy plus trastuzumab in postmenopausal women with locally advanced or metastatic HR+/HER2+ breast cancer. Participants must have received at least two lines of HER2-directed therapy prior to enrollment, including trastuzumab emtansine (T-DM1) in any disease setting. Patients must not have received prior treatment with CDK4/6 inhibitors. The study is currently ongoing. The PATRICIA trial is an ongoing multicenter phase II trial evaluating the use of the combination of palbociclib plus trastuzumab, with or without letrozole, in post-menopausal women with HER2-positive advanced or metastatic breast cancer previously treated with chemotherapy and trastuzumab. The study includes three treatment arms. Arm A includes patients with ER−/HER2+ disease who will receive palbociclib and traztuzumab. Arms B2 and B2 include patients with ER+/HER2+ disease who will receive palbiciclib plus trastuzumab and palbocilcib/traztuzumab/letrozole, respectively. Another study evaluates the use of tucatinib, a novel HER2-targeted tyrosine kinase inhibitor, in combination with palbociclib and letrozole as a first- or second-line therapy option for patients with HER2+/ER+ metastatic breast cancer.

### P13K inhibitors

#### Mechanism of action

As mentioned previously, the P13K-AKT-mTOR pathway is a frequently activated signaling pathway in breast cancer. The P13K pathway may be involved in resistance to cancer therapies, including endocrine therapy, chemotherapy, and targeted drugs. Inhibition of P13K may help restore sensitivity to other therapies when use in combination regimens. A number of P13K inhibitors are under investigation for treatment of several malignancies, including breast cancer (Table 2).

**Table 2:** Summary of trials combining endocrine therapy with PI3K inhibitors in patients with metastatic hormone receptor-positive breast cancer

Agent	ET	Setting	Trial
Alpelisib	Fulvestrant	After progression on AI	SOLAR-1; NCT02437318
Buparlisib	Fulvestrant	After progression on AI	BELLE-2; NCT01610284
Buparlisib	Fulvestant	After progression on mTOR inhibitor	BELLE-3; NCT01633060
Pictilisib	Fulvestrant	After progression on AI	FERGI; NCT01437566
Pictilisib + Palbociclib	Fulvestrant	After progression on AI	PASTOR; NCT02599714
Gedatolisib + Palbociclib	Fulvestrant or Letrozole	3 arms: • No prior endocrine therapy • Progression on or after 1 line of endocrine therapy, no prior CDK inhibitor therapy • Progression after 1 or 2 prior endocrine therapies, following prior CDK inhibitor therapy	NCT02684032

*ET* endocrine therapy, *AI* aromatase inhibitor

#### Alpelisib

Alpelisib has demonstrated promising early efficacy in studies, both as a single agent and in combination with fulvestrant [32–34]. Data presented by Juric et al. demonstrated an improved disease control rate and clinical benefit rate in patients with P13KCA-mutations, compared with no response in those with wild-type tumors. The SOLAR-1 trial is an ongoing phase III study of the use of alpelisib combined with fulvestrant in men and postmenopausal women with ER-positive/HER2-negative breast cancer which progressed on or after treatment with an aromatase inhibitor.

#### Buparlisib

Buparlisib is a pan-P13K inhibitor that inhibits all four of the class 1 P13K isoforms [35]. The BELLE-2 trial was a phase III study evaluating the use of buparlisib plus fulvestrant in post-menopausal women with hormone receptor-positive, HER2-negative advanced or metastatic breast cancer which had progressed on an aromatase inhibitor [36]. A total of 1147 women were randomized to receive either a buparlisib/fulvestrant combination or fulvestrant monotherapy. There was a significant improvement in median PFS observed in the buparlisib arm compared with the fulvestrant arm (6.9 vs 5.0 months). Among patients with known P13K pathway status, median PFS in the combination and control arm was 6.8 and 4.0 months, respectively. There was no significant difference in PFS between the treatment arms in patients without P13K-mutations. Overall survival data was immature at the time of study assessment. Serious adverse events occurred in 23% of patients treated in the bupalisib arm compared with 16% in the control arm, the most common of which were elevations in AST and ALT and hyperglycemia.

The BELLE-3 trial evaluated the use of combination treatment with buparlisib and fulvestrant in patients with HR-positive/HER2-negative MBC who had progressed on or after treatment with an mTOR inhibitor [37]. Similar to the BELLE-2 study, there was an improvement in median PFS in the bupalisib arm compared with the control arm (3.9 vs 1.8 months). Toxicity data was similar to that of the BELLE-2 study.

#### Pictilisib

The FERGI trial was a two-part phase 2 study of the use of pictilisib plus fulvestrant in post-menopausal women with ER+/HER2− advanced or metastatic breast cancer resistant to treatment with an aromatase inhibitor [38]. Patients were randomized to receive either pictilisib plus fulvestrant or placebo plus fulvestrant. Part 1 of the study included patients with and without P13K mutations, while part 2 only included patients with P13K mutations. No difference in PFS was found between treatment arms in either part 1 or part 2 of the study. This lack of improvement in PFS may in part be due to significant toxicity associated with pictilisib use, leading to many patients not receiving the full dose of the drug until progression. Forty (45%) of patients in part 1 had dose modifications. The pictilisib dose was reduced for part 2 of the study; however, 17% of patients still required dose reductions due to toxicity.

#### Taselisib

Taselisib is an investigational P13K being studied in combination with fulvestrant in patients with advanced or metastatic breast cancer who have progressed or recurred during or after treatment with an aromatase inhibitor (NCT02340221).

#### Combined CDK4/6 and mTOR inhibition

The interaction between the CDK4/6 and P13K/mTOR/AKT pathways is thought to play an important role in endocrine receptor-positive breast cancer. Studies show that CDK4/6 resistant cell lines remain sensitive to mTORC 1/2 inhibition, suggesting that combining these therapies may be an option for patient who has relapsed while on CDK4/6 therapy. Studies are being conducted studying novel combinations of P13K and CDK4/6 inhibitors with endocrine therapy [39].

The PASTOR trial is a phase I/II multicenter trial of the combination of vistusertib, palbociclib, and fulvestrant in postmenopausal patients with advanced or metastatic endocrine receptor-positive breast cancer. There is also an ongoing phase Ib study of the use of gedatolisib, a potent dual P13K/mTOR inhibitor, in combination with palbociclib/fulvestrant and with palbociclib/letrozole [40].

#### Summary

There are multiple ongoing trials combining PI3K inhibitors with other agents, such as CDK4/6 inhibitors and endocrine therapy. Future therapy may evaluate the use of these drugs with inhibitors to other pathways, such as STAT3, MYC, MEK, and PARP.

### Third- and fourth-line therapies

High-dose estrogens and progestins can be considered as late-line options for therapy in metastatic hormone-receptor positive breast cancer. Megesterol acetate and medroxyprogesterone are progestins with activity in metastatic breast cancer. A study of postmenopausal women with locally advanced or metastatic breast cancer who had progressed on first-line tamoxifen demonstrated an overall response rate of 25% with megesterol acetate and 43% with medroxyprogesterone [41]. Median progression-free survival was 15 months for megesterol and 10 months for medroxyprogesterone. A dose-escalation trial of megesterol acetate demonstrated a higher toxicity rate without any improvement in effectiveness with the higher doses [42]. Therefore, the 160 mg daily dose is generally used. Estrogen compounds have also been used in the management of metastatic breast cancer. A study of 32 patients with endocrine therapy-resistant disease treated with diethylstilbestrol (DES) yielded an objective response rate of 31% [43]. Patients had received an average of 4 prior endocrine therapies and 1 prior chemotherapy. A phase II study evaluating the use of 6 versus 30 mg of estradiol in patients with advanced breast cancer showed that the lower dose provided a similar clinical outcomes as the 30 mg dose. The lower dose was also associated with fewer adverse events.

### Optimal sequence of therapy

**Fig. 1:**
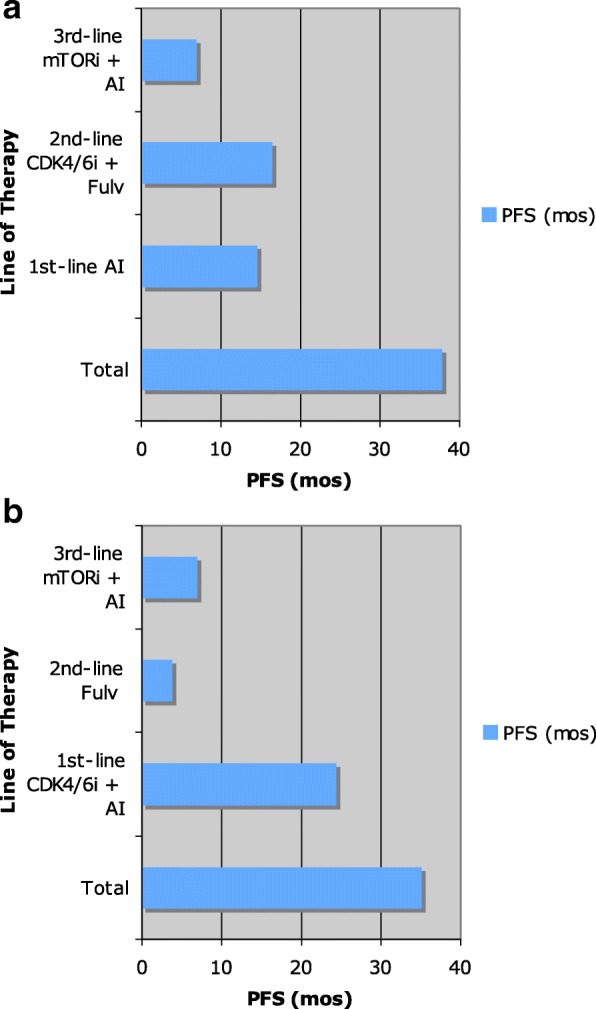
Progression-free survival (PFS) using endocrine therapy. **a** The PFS when aromatase inhibitors (AIs) are used first line. **b** The PFS with the use of front-line CDK4/6 inhibitors

There are many options in the sequencing of therapy for endocrine receptor-positive, metastatic breast cancer in post-menopausal women. Although first-line treatment with a CDK/4/6 inhibition has significant improvement in PFS, the total PFS is similar regardless of the sequencing (Fig. 1a, b). Treatment decisions should be based on medical comorbidities, prior adjuvant therapies, and disease-free interval [44]. First-line treatment with an aromatase inhibitor or fulvestrant are still viable options and offer a PFS of 14 and 16.6 months, respectively. Frontline use of the combination of a CDK4/6 inhibitor with an aromatase inhibitor, such as palbociclib/letrozole and ribociclib/letrozole, offer a greater than 24-month PFS. Subsequent-line therapies include the use of palbociclib or abemaciclib with fulvestrant, the combination of everolimus with exemestane, and single-agent abemaciclib. The use of PI3K-inhibitors both single-agent and in combination with fulvestrant are being studied for use in patients with endocrine-resistant disease. Immunotherapy and CAR-T therapy are also being explored as other options of treatment. The use of biomarkers, including ESR1 mutation, and genomic profiling may provide useful future tools to direct therapy.

### Future directions

There are several ongoing studies of the use of combinational treatments including PD-1 and PD-L1 inhibitors in breast cancer treatment [45, 46]. The JPCE trial included 28 patients with hormone receptor-positive, HER2-negative metastatic breast cancer treated with abemaciclib and pembrolizumab. The PR rate at 16 weeks was 14.3%, and the overall response rate at 16-week analysis was 14.3%. Another ongoing trial assesses the combination of pembrolizumab, letrozole, and palbociclib in postmenopausal patients with metastatic estrogen receptor-positive breast cancer who did not respond to letrozole and palbocilcib.

There are multiple ongoing studies of the use of CAR-T therapy for patients with breast cancer. Another means of treatment being evaluated for use in solid tumors are bispecific antibodies [47, 48].

Utomilumab (PF-05082566) is a fully human IgG2 agonist monoclonal antibody that binds to 4-1BB/CD137. This binding induces T-cell proliferation, production of cytokines, and inhibition of tumor growth in severely compromised immunodeficient xenograft models [49, 50]. The drug is currently being studied in combination with anti-PD1 and anti-PD-L1 agents in the treatment of solid tumors.

Entinostat is an oral benzamide derivative that acts by selectively inhibiting class I and IV histone deacetylase. The ENCORE II study was a phase II trial evaluating the use of entinostat and exemestane in patients with advanced hormone receptor-positive breast cancer that had progressed on a prior non-steroidal aromatase inhibitor. This study demonstrated a significant improvement in progression-free and overall survival. E2112 is a double-blind phase III study evaluating the use of exemestane plus entinostat/placebo in the same population and is currently ongoing [51].

The PALLAS study is an ongoing trial evaluating the use of adjuvant palbociclib in combination with standard adjuvant endocrine therapy in patients with early hormone receptor-positive, HER2-negative breast cancer. The use of CDK4/6 inhibitors as part of adjuvant therapy will have a significant impact on future therapy options and the sequencing of their use in the metastatic and recurrent settings.

The PEARL and POLARIS studies are other ongoing studies of palbociclib.

These studies may yield additional therapeutic options in the future for patients with metastatic hormone receptor-positive breast cancer.

## Conclusions

Endocrine therapy forms the cornerstone of treatment for advanced-stage hormone receptor-positive breast cancer. The development of endocrine resistance has prompted the development of a multitude of novel therapeutic options, including CDK4/6, mTOR, and PI3K inhibitors. There are many additional treatments currently in development. The optimal sequencing of therapy can be complicated and depends on different factors, including prior adjuvant therapy, disease-free interval, side effects, and patient quality of life.

## Abbreviations

AI: Aromatase inhibitor
CDK4/6: Cyclin-dependent kinase 4/6
CR: Complete response
DLT: Dose-limiting toxicity
ER: Estrogen receptor
HR: Hazard ratio
ORR: Overall response rate
PFS: Progression-free-survival
SERD: Selective estrogen receptor downregulator
